# Neonate screening for glucose-6-phosphate dehydrogenase deficiency in South Kivu, eastern Democratic Republic of Congo

**DOI:** 10.1186/s12887-026-07093-x

**Published:** 2026-06-02

**Authors:** Isia Nanci Francisca, Bisimwa Balakula Ghislain, Amani Ngaboyeka Gaylord, Boemer Francois, Bours Vincent, Battisti Oreste, Mbusa Kambale Richard

**Affiliations:** 1 Department of Pediatric, Hôpital Provincial Général de Référence de Bukavu, Bukavu, Democratic Republic of the Congo; 2 Faculty of Medecine, Université Catholique de Bukavu, Bukavu, Democratic Republic of the Congo; 3 Regional School of Public Health, Université Catholique de Bukavu, Bukavu, Democratic Republic of the Congo; 4 Institut Supérieur des Techniques Médicales de Kanyamulande, Walungu, Democratic Republic of the Congo; 5 Biochemical Genetics Laboratory of the University Hospital of Liège, Liège, Belgium; 6 Clinical Department of science, Faculty of Medecine, University of Liège, Liège, Belgium; 7 Institut Supérieur des Techniques Médicales de Bukavu, Bukavu, Democratic Republic of the Congo; 8 Center for Tropical Diseases and Global Health, Université Catholique de Bukavu, Bukavu, Democratic Republic of the Congo

**Keywords:** G6PD deficiency, Neonatal screening, Hyperbilirubinemia, Democratic Republic of the Congo

## Abstract

**Background:**

Glucose-6-phosphate dehydrogenase (G6PD) deficiency is a common inherited enzymatic disorder and an important risk factor for severe neonatal hyperbilirubinemia. In the Democratic Republic of the Congo (DRC), neonatal data on G6PD deficiency remain limited. This study aimed to screen for G6PD deficiency among neonates in South Kivu, eastern DRC.

**Methods:**

A cross-sectional hospital-based study was conducted from April to August 2022 in three maternity wards in South Kivu. Live-born neonates whose parents provided informed consent were screened within one hour of birth. G6PD activity was measured on dried blood spots using the fluorescent spot test. G6PD deficiency was defined as enzyme activity < 30% of normal activity. Prevalence estimates with 95% confidence intervals (CI) were calculated, and associations were assessed using bivariate analysis.

**Results:**

Among 1,188 neonates screened, four had doubtful results. Among the 1,184 neonates with valid results, 73 were G6PD deficient, corresponding to an overall prevalence of 6.2% (95% CI: 4.9–7.7). Prevalence ranged from 2.9% to 7.7% across study sites, with no significant difference between maternity wards (*p* = 0.11). G6PD deficiency was more frequent among males than females (10.1% vs. 1.5%), and male sex was significantly associated with deficiency (odds ratio 7.49; 95% CI: 3.56–15.77).

**Conclusion:**

Neonatal G6PD deficiency is relatively common in South Kivu and exceeds thresholds commonly proposed for targeted neonatal screening. These findings support consideration of neonatal G6PD screening strategies in the DRC.

**Supplementary Information:**

The online version contains supplementary material available at 10.1186/s12887-026-07093-x.

## Introduction

Glucose-6-phosphate dehydrogenase (G6PD) deficiency is an X-linked inherited enzymatic disorder caused by mutations in the *G6PD* gene [1]. The enzyme plays a critical role in the pentose phosphate pathway by generating nicotinamide adenine dinucleotide phosphate (NADPH), which is essential for maintaining redox balance in erythrocytes [1]. As red blood cells rely exclusively on this pathway for NADPH production, G6PD deficiency results in impaired antioxidant defense and increased susceptibility to oxidative stress, leading to hemolysis, anemia, and hyperbilirubinemia [2].

G6PD deficiency is the most common inherited red blood cell enzymopathy worldwide, affecting an estimated 400–500 million individuals [3]. The distribution of G6PD deficiency is geographically heterogeneous, with the highest prevalence reported in sub-Saharan Africa, followed by the Middle East and Southeast Asia [4]. In the Democratic Republic of the Congo (DRC), population-based data remain limited; however, a community study among rural children aged 6–59 months reported a prevalence of 28.3%, suggesting a substantial burden in certain settings [5].

The high prevalence of G6PD deficiency in malaria-endemic regions has led to the hypothesis of a selective protective effect against malaria, although the biological mechanisms underlying this association remain incompletely understood [6–8]. Despite this potential evolutionary advantage, G6PD deficiency is a well-established risk factor for neonatal morbidity, particularly early-onset unconjugated hyperbilirubinemia [9]. Severe hyperbilirubinemia may progress to acute bilirubin encephalopathy and kernicterus if not promptly identified and managed.

Neonatal screening is a cornerstone of public health strategies aimed at the early detection of inherited disorders [10]. Although universal neonatal screening for G6PD deficiency has not been globally adopted, expert consultations and technical guidance from the World Health Organization (WHO) recommend targeted or population-based screening in settings where the prevalence of G6PD deficiency is high (≥ 3–5% among males), particularly in contexts with substantial neonatal jaundice-related morbidity and mortality and limited access to bilirubin testing and effective phototherapy [11–13].

In the DRC, these criteria are met. National molecular studies have demonstrated the presence of *Plasmodium vivax* malaria across the country, with implications for primaquine-based radical cure strategies that require prior G6PD testing because of the risk of hemolysis [14, 15]. At the same time, neonatal hyperbilirubinemia remains a preventable cause of severe morbidity and mortality, and access to timely bilirubin measurement and reliable phototherapy remains limited in many health facilities [16, 17]. The convergence of these factors provides a strong public health rationale for neonatal G6PD screening in the DRC. Accordingly, this study aimed to screen for G6PD deficiency among neonates in South Kivu, eastern DRC.

## Materials and methods

### Study design and setting

A cross-sectional, hospital-based study was conducted between April and August 2022 in three maternity wards in South Kivu Province, eastern DRC. These included one urban facility, Hôpital Général de Référence de Bukavu (HPGRB), and two rural facilities, Hôpital Général de Référence de Miti-Murhesa (HGRM) and Hôpital Général de Référence de Nyantende (HGRN).

HPGRB is one of the main referral hospitals in the city of Bukavu and records approximately 2,500 deliveries annually. HGRM is located in the Kabare administrative zone, about 31 km north of Bukavu, and reports approximately 1,500 deliveries per year. HGRN is also situated in the Kabare administrative zone, approximately 10 km south of Bukavu, with an estimated annual delivery volume of 2,000. The three maternity wards were selected based on demographic and epidemiological representativeness, geographic accessibility, and logistical feasibility.

### Inclusion and exclusion criteria

All live-born neonates whose parents or legal guardians provided written informed consent were eligible for inclusion. Neonates were screened within an hour of birth. Exclusion criteria included stillbirths, neonates with major congenital malformations, neonates in critical condition requiring admission to a neonatal intensive care unit (including respiratory distress, shock, or hypoxic–ischemic encephalopathy), and neonates whose parents declined participation.

### Sample size determination

The sample size was calculated using the formula $\:n=\frac{{z}^{2}\mathrm{p}\mathrm{q}}{{d}^{2}}$ (z = 1.96; *p* = 0.075, q = 1-p, d = 0.015), considering a 95% confidence interval with an absolute precision of 1.5% and assuming that 7.5% of newborns have G6PD deficiency, based on G6PD deficiency frequency in Sub-Saharan Africa [4]. The minimum required sample size was 1,185 neonates. To account for potential pre-analytical errors, particularly those related to blood sample collection, storage, and enzyme degradation that could result in false-negative results, a 10% increase was applied. The final target sample size was therefore set at 1,300 neonates.

### Data and sample collection

Umbilical cord blood samples were collected at birth into Ethylene Diamine Tetra-Acetic (EDTA) tubes and gently inverted several times to prevent clotting. Cord blood sampling is widely used in neonatal screening research using dried blood spots. This sampling approach has been used in several neonatal screening studies using dried blood spots and allows standardized preparation of filter paper samples for enzymatic analysis. Using a calibrated micropipette, six drops of whole blood were applied to pre-labelled Whatman 903 S&S filter paper cards (GE Healthcare). The cards were air-dried horizontally for at least four hours at room temperature, protected from direct sunlight.

After drying, each card was individually sealed in an airtight plastic bag containing desiccant to minimize moisture exposure and enzyme degradation. Samples collected at HGRM and HGRN were transported within 24 h to the HPGRB laboratory, where identical handling and storage procedures were applied.

All dried blood spot cards were stored at room temperature and subsequently shipped to the genetic biochemistry laboratory of the University Hospital of Liège (Belgium) for analysis. For the revised analysis, samples for which the interval between collection and shipment exceeded 15 days were excluded in order to minimize potential misclassification related to enzyme degradation during prolonged pre-analytical storage. This approach was implemented in response to concerns regarding potential enzyme degradation during prolonged storage of dried blood spots. The results presented in this study therefore reflect analyses restricted to samples meeting this stricter pre-analytical criterion.

G6PD activity was assessed using the Fluorescent Spot Test method [18, 19]. Briefly, glucose-6-phosphate and nicotinamide adenine dinucleotide phosphate (NADP⁺) substrates were added to eluates from dried blood spots. Enzymatic activity was determined by measuring the generation of NADPH after 10 min at ambient temperature using fluorescence detection (excitation at 360 nm; emission at 450 nm) on a Victor X5 device (Revvity, formerly PerkinElmer). G6PD activity (%) was calculated against calibration samples generated from ten whole-blood samples with normal G6PD activity spotted on dried blood spot, with the mean activity of these samples defined as 100%. No correction for hemoglobin concentration was applied. G6PD deficiency was defined as enzyme activity below 30% of normal activity [20]. Results were ultimately reported in a bimodal manner as deficient or not deficient.

Sociodemographic data (sex, gestational age, and region of origin) and anthropometric measurements (birth weight, length, and head circumference) were collected using standardized procedures.

### Statistical analysis

Data were analyzed using Stata software version 16 (StataCorp, College Station, TX, USA). G6PD status was the dependent variable and was categorized as deficient (< 30% enzymatic activity) or normal (≥ 30%) [20]. Continuous variables were summarized as medians with interquartile ranges (IQRs), while categorical variables were presented as frequencies and percentages.

Comparisons between categorical variables were performed using the χ² test or Fisher’s exact test, as appropriate. The Mann–Whitney U test was used to compare non-normally distributed continuous variables. Factors associated with G6PD deficiency were evaluated using bivariate analysis, with odds ratios (OR) and 95% confidence intervals (95% CI) calculated. Statistical significance was set at *p* < 0.05.

### Ethical considerations

The study was conducted in accordance with the principles of the Declaration of Helsinki. Ethical approval was obtained from the Ethics Committee of the Université Catholique de Bukavu (Approval No. UCB/CIES/NC/005/2022). Written informed consent was obtained from parents or legal guardians prior to enrollment. Parents of neonates with confirmed G6PD deficiency were contacted and informed of the diagnosis and received an educational brochure detailing G6PD deficiency, known hemolysis triggers, and clinical signs requiring medical consultation.

## Results

A total of 1,188 newborns were included in the study, of whom 548 were from urban areas and 640 from rural areas. A slight male predominance was observed (645 males and 543 females; sex ratio 1.19). Most newborns were born at term and had birth weights appropriate for gestational age. Additional demographic and clinical characteristics are summarized in Table 1.

**Table 1:** Demographic characteristics of the study participants

Characteristics	Total (*n* = 1188)	HPGRB *n* = 548	HGRM *n* = 238	HGRN *n* = 402	*P*-value
Male sex	645 (54.3)	295 (53.8)	135 (56.7)	215 (53.5)	0.698
Preterm	78 (6.6)	65 (11.9)	2 (0.8)	11 (2.7)	< 0.001
Twins	42 (3.5)	38 (6.9)	2 (0.8)	2 (0.5)	< 0.001
Small for gestational age	70 (5.9)	41 (7.5)	16 (6.7)	13 (3.2)	0.019
Large for gestational age	61 (5.1)	32 (5.8)	6 (2.5)	23 (5.7)	0.124
Birth weight (kg), median (IQR)	3.2 (2.8–3.5)	3.2 (2.8–3.5)	3.1 (2.8–3.4)	3.2 (2.9–3.5)	0.031
Birth length (cm), median (IQR)	49 (48–50)	49 (48–50)	50 (50–51)	49 (47–50)	< 0.001
Birth HC (cm), median (IQR)	35 (34–36)	35 (34–36)	35 (35–35)	34 (33–35)	< 0.001

*G6PD* Glucose-6-phosphate dehydrogenase, *HC* Head circumference, *HGRM* Hôpital Général de Référence de Miti-Murhesa, *HGRN* Hôpital Général de Référence de Nyantende, *HPGRB* Hôpital Provincial Général de Référence de Bukavu, *IQR* Interquartile range

Among the 1,188 newborns screened, 4 (0.3%) had doubtful results and were subsequently called back for further testing. These four samples were excluded from prevalence calculations. Among the 1,184 newborns with valid results, 73 were found to be G6PD deficient, corresponding to an overall prevalence of 6.2% (95% CI: 4.9–7.7). The prevalence varied across study sites, ranging from 2.9% in HGRM to 7.7% in HGRN, but the difference between sites was not statistically significant (*p* = 0.11) (Table 2).

**Table 2:** Frequency of G6PD deficiency among newborns, overall and stratified by study site

Study site	*n* / *N*	Frequency (%)	95% CI (%)	*p*
HPGRB	35 / 548	6.4	4.6–8.8	0.11
HGRM	7 / 238	2.9	1.4–6.0	
HGRN	31 / 402	7.7	5.5–10.7	
Overall	73 / 1184	6.2	4.9–7.7	

*CI* Confidence intervals, *G6PD* Glucose-6-phosphate dehydrogenase, *HGRM* Hôpital Général de Référence de Miti-Murhesa, *HGRN* Hôpital Général de Référence de Nyantende, *HPGRB* Hôpital Provincial Général de Référence de Bukavu

**Fig. 1:**
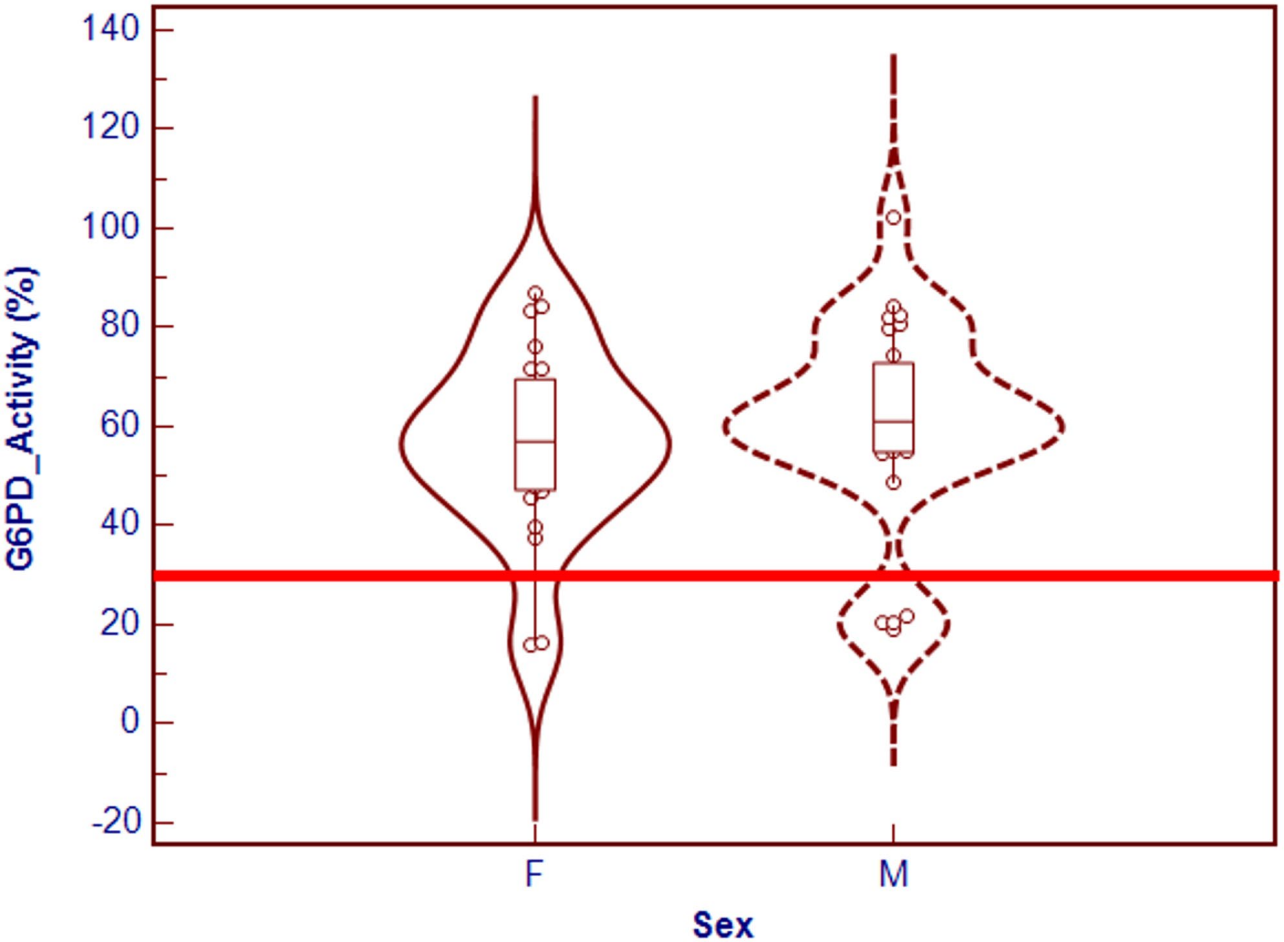
Distribution of quantitative G6PD enzymatic activity according to sex among screened neonates

In bivariate analysis, sex was strongly associated with G6PD deficiency (Table 3). The prevalence of G6PD deficiency was 10.1% among male neonates compared with 1.5% among females. Male neonates had significantly higher odds of G6PD deficiency than females (OR = 7.49; 95% CI: 3.56–15.77). Figure 1 presents the distribution of quantitative G6PD enzymatic activity according to sex, highlighting the greater proportion of values below the deficiency threshold among male neonates.

**Table 3:** Bivariate analysis of risk factors associated with G6PD deficiency

Characteristics	% G6PD deficient	OR (95%CI)
Sex		
Male (*n* = 645)	10.1	7.49 (3.56–15.77) *
Female (*n* = 543)	1.5	1
Residence area		
Rural** (*n* = 640)	5.9	0.93 (0.58–1.49)
Urban (*n* = 548)	6.4	1

*G6PD* Glucose-6-phosphate dehydrogenase, *OR* Odds ratio

* *p*< 0.05

** Rural residence included Hôpital Général de Référence de Miti-Murhesa (HGRM) and Hôpital Général de Référence de Nyantende (HGRN)

The dashed horizontal line represents the threshold defining G6PD deficiency (< 30% of normal activity).

## Discussion

In this hospital-based neonatal screening study conducted in South Kivu, eastern DRC, the prevalence of G6PD deficiency was 6.2%. This prevalence indicates that G6PD deficiency represents an important neonatal health concern in this setting. Our findings contribute important neonatal data from a context where such evidence remains limited.

Data on neonatal G6PD deficiency in the DRC are scarce. A previous mother–newborn cohort study from another region of the country reported a lower prevalence of approximately 2%, whereas community-based studies among older children have described substantially higher frequencies, sometimes exceeding 20% [16, 21]. These differences likely reflect variation in study populations, age groups, geographic settings, and diagnostic approaches, as phenotypic screening tests may underestimate deficiency—particularly among heterozygous females—compared with genotypic methods.

Across Central Africa, reported prevalences of G6PD deficiency generally range from 5% to 15%, depending on study design and population characteristics. Estimates from countries such as Cameroon and Equatorial Guinea are comparable to, or slightly higher than, those observed in the present study, particularly among males [4, 22]. Our findings therefore fall within the expected regional range.

The prevalence observed in this study exceeds the 3–5% threshold commonly cited by the WHO as supporting targeted or population-based neonatal G6PD screening in high-risk settings [10–12]. In the context of the DRC, these findings have direct implications for national newborn health policy. First, G6PD deficiency is a well-established risk factor for early-onset severe neonatal hyperbilirubinemia, a major contributor to preventable neonatal morbidity and mortality in the country [22, 23]. Importantly, G6PD deficiency is clinically relevant because it may predispose to hemolysis under oxidative stress; however, the present study did not assess hemolytic episodes, and this reference is provided solely to contextualize the importance of early identification. Second, the documented presence of *P. vivax* malaria in the DRC has implications for malaria elimination strategies that rely on 8-aminoquinoline drugs, which require prior knowledge of G6PD status to avoid drug-induced hemolysis [14, 24]. Third, limited access to bilirubin measurement and effective phototherapy in many Congolese health facilities further amplifies the potential benefit of early identification of G6PD-deficient neonates.

Taken together, these findings support the integration of neonatal G6PD screening into a phased national newborn screening strategy in the DRC. In resource-limited settings such as eastern DRC, implementing point-of-care (POC) G6PD testing could represent a practical alternative to centralized laboratory assays. Several POC devices, including CareStart™ G6PD and STANDARD™ G6PD tests, have shown promising performance in field conditions and could facilitate decentralized neonatal screening programs. An initial approach could prioritize high-prevalence provinces, referral maternity hospitals, and settings with limited neonatal jaundice management capacity. Integration with existing maternal and child health services, combined with parental education and clear referral pathways, could enhance feasibility and sustainability while contributing to reductions in preventable neonatal morbidity and mortality.

This study has several strengths. It is among the first neonatal G6PD screening studies conducted in eastern DRC, includes a large sample size, and draws data from both urban and rural maternity wards, enhancing internal validity. Standardized sample collection procedures and centralized laboratory analysis further strengthen the reliability of the findings.

However, several limitations should be acknowledged. First, the hospital-based design may limit generalizability to home births and other regions of the country. Second, confirmatory genotypic testing and longitudinal follow-up to assess clinical outcomes such as neonatal jaundice were beyond the scope of the study. In the context of external quality assessment, NSQAP samples are typically shipped at room temperature and stored at − 20 °C upon arrival in the laboratory. The transport duration generally ranges from a few days to two weeks. This practice suggests that short-term storage at ambient temperature during transport may still allow reliable measurement of G6PD activity, although enzyme degradation remains a recognized pre-analytical concern. Third, according to Fig. 4 of the study by Chamchoy et al. [23], G6PD activity may decrease during prolonged storage of dried blood spots at ambient temperature. In the present study, to minimize this potential bias, we restricted the main analysis to samples for which the interval between collection and shipment did not exceed 15 days. Under these conditions, the likelihood of substantial enzyme degradation is expected to be limited. Experimental studies have nevertheless shown that prolonged storage of dried blood spots for several months may lead to progressive reduction in measurable G6PD activity. For example, Chamchoy et al. reported a decline in activity from approximately 60–70% to about 40% after one to four months of storage at room temperature with desiccant [23]. Such degradation could theoretically increase the risk of false-positive classification, particularly among heterozygous females whose enzymatic activity typically ranges between 30% and 80% [25]. Consequently, storage of dried blood spot for two months at ambient temperature may eventually have led to false-positive classifications in this group.

## Conclusion

This study found a moderate but clinically relevant prevalence of neonatal G6PD deficiency in South Kivu, eastern DRC. The observed prevalence exceeds commonly recommended thresholds for targeted neonatal screening and highlights the importance of early identification in settings with a high burden of neonatal jaundice and malaria. Integrating neonatal G6PD screening into existing maternal and newborn health services could support early risk stratification, parental counseling, and safer malaria treatment strategies. Future multicenter studies including molecular characterization of G6PD variants would help guide national implementation strategies. To guide national policy and implementation, large-scale, multicenter studies are needed to evaluate the feasibility and cost-effectiveness of program scale-up and to characterize circulating G6PD variants, thereby clarifying the clinical and public health benefits of early newborn identification in the DRC.

## Supplementary Information

Supplementary Material 1.

## Data Availability

The datasets used and/or analysed during the current study are available from the corresponding author on reasonable request **.**.
